# Engaging Broader Stakeholders to Accelerate Group A *Streptococcus* Vaccine Development

**DOI:** 10.3390/vaccines13070734

**Published:** 2025-07-07

**Authors:** Dechuan Kong, Hao Pan, Huanyu Wu, Jian Chen

**Affiliations:** 1 Department of Communicable Diseases Control and Prevention, Shanghai Municipal Center for Disease Control and Prevention, Shanghai 200336, China; 2 Office of the Director, Shanghai Municipal Center for Disease Control and Prevention, Shanghai 200336, China

**Keywords:** Group A *Streptococcus*, GAS, vaccine, M protein, multivalent, J8

## Abstract

Group A *Streptococcus* (GAS) imposes a significant global health burden across all age groups, annually causing over 600 million cases of pharyngitis and more than 18 million severe invasive infections or sequelae. The resurgence of scarlet fever globally and streptococcal toxic shock syndrome (STSS) outbreaks in Japan have brought GAS infections back into the spotlight as a pressing global health concern. Unfortunately, no licensed vaccine against GAS is yet available for clinical use. Our comprehensive review examines the developmental history of GAS vaccines, outlining the research trajectory from early inactivated vaccines to contemporary multivalent, conjugate, multi-antigen, and mRNA-based vaccine platforms. It systematically analyzes clinical trial outcomes of GAS vaccines, highlighting recent advances in both M protein-based and non-M protein vaccine candidates while summarizing promising target antigens. The review concludes with critical strategies to accelerate vaccine commercialization, including enhanced investment in research and development, expanded collaborations, leveraging advanced vaccine technologies, streamlined clinical trials, and strengthened public health advocacy. This review critically evaluates the current evidence and future prospects in GAS vaccine development, emphasizing innovative strategies and engaging broader stakeholders to accelerate GAS vaccine development.

## 1. Introduction

Before the COVID-19 pandemic, the resurgence of scarlet fever in 2011 drew global attention to Group A *Streptococcus* (GAS) [1,2,3]. Following the pandemic, streptococcal toxic shock syndrome (STSS) incidents in Japan in 2024 have once again brought GAS into the spotlight for researchers [4,5]. In April 2025, JAMA reported a significant increase in invasive GAS infections across 10 U.S. states [6]. Let us take another look at the “criminal record” of GAS. Scientists discovered GAS more than 100 years ago. GAS poses a substantial global disease burden, impacting individuals of all ages. GAS is responsible for a wide range of illnesses, from mild infections such as pharyngitis, scarlet fever and impetigo to severe invasive diseases like necrotizing fasciitis and STSS. Additionally, post-infectious complications such as acute rheumatic fever (ARF) and rheumatic heart disease (RHD) contribute significantly to long-term morbidity and mortality.

According to estimates, GAS causes over 600 million cases of pharyngitis annually, with more than 18 million people suffering from severe invasive diseases or their sequelae. Rheumatic heart disease alone accounts for approximately 233,000 deaths each year globally, disproportionately affecting children and young adults in underprivileged regions [7,8,9]. Invasive GAS diseases, though less common, carry high mortality rates, often exceeding 25%, even with advanced medical interventions [7,10]. The economic and social impact of GAS infections is profound [11]. Recurrent pharyngitis contributes to school absenteeism in children and work disruption for caregivers, while invasive diseases impose a significant healthcare burden due to prolonged hospital stays and complex treatments.

Beyond the considerable public health burden imposed by GAS infections, the escalating antimicrobial resistance in GAS has emerged as a critical concern, presenting formidable therapeutic challenges [12]. GAS remains highly susceptible to penicillin, making it the first-line treatment for GAS infections, including pharyngitis and invasive diseases [12,13]. However, resistance to macrolides has become a growing concern, particularly in Asia, where resistance rates reach 50–60%, primarily due to the spread of *ermB* (methylase-mediated) and *mefA* (efflux pump) resistance genes [12,13,14,15]. Additionally, tetracycline resistance is widespread in developing countries, while clindamycin resistance often exhibits inducible resistance, requiring D-test confirmation [12,16,17,18,19].

This raises a pressing question: beyond emerging infectious diseases like COVID-19, why does this ancient bacterium continue to pose a severe threat to public health? Experts in the field might argue that the lack of an effective vaccine remains a critical factor. However, the next question arises: why, after so many years, do we still not have a usable *GAS* vaccine? Indeed, this is a pivotal question.

In this review, we searched PubMed/MEDLINE for GAS vaccine studies and ClinicalTrials.gov for clinical trials on *GAS*/HIV/dengue vaccines, with publication dates up to 27 April 2025. The final search was conducted on 29 April 2025. First, we screened titles and abstracts for inclusion/exclusion based on our core research themes. For pivotal milestone publications, we conducted full-text critical appraisals and performed forward/backward citation tracking. Additionally, we supplemented our data by retrieving relevant information from authoritative sources such as the WHO website. This review delves into the history of *GAS* vaccine development, summary, and analysis of clinical trial trends, exploring potential approaches to accelerate *GAS* vaccine development. Our findings aim to offer actionable insights and evidence-based recommendations to facilitate the successful development and timely commercialization of effective *GAS* vaccines.

## 2. The Development of GAS Vaccines

**Figure 1:**
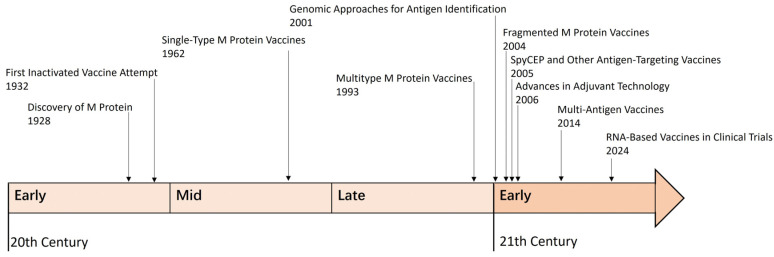
A brief history of GAS vaccine development.

The development of GAS vaccines has been a complex journey spanning over a century, driven by advances in microbiology, immunology, and vaccine technology (Figure 1).

Early efforts in the 20th century focused on whole-cell vaccines, which were abandoned due to severe side effects, including autoimmune reactions like rheumatic fever, caused by cross-reactivity between GAS antigens and human tissues [20,21]. The discovery of the M protein, a major virulence factor with over 200 serotypes, highlighted the challenge of antigenic diversity [22]. Subsequent research shifted toward subunit vaccines targeting conserved regions of the M protein to avoid triggering autoimmune responses, leading to the development of multivalent vaccines that could cover multiple serotypes [23,24,25,26,27,28]. Non-M protein antigens, such as SpyCEP, SLO, and C5a peptidase, were also identified as promising targets due to their conserved nature [29].

In the 21st century, advances in genomics and reverse vaccinology enabled the identification of highly conserved and immunogenic antigens, while molecular techniques allowed the creation of multi-epitope fusion vaccines [28,30]. The field is witnessing a technological emergence in *GAS* mucosal vaccines [31], marked by advancing adjuvant systems and parallel development of both multi-antigen vaccines (e.g., SpyCEP) and mRNA vaccine platforms [27,32,33]. Emerging platforms like mRNA technology have further enhanced vaccine development, offering robust immune responses and scalable production.

## 3. Clinical Trials

Current strategies employing structure-guided antigen design, novel adjuvants, and multivalent platforms show particular promise (Table 1). The most advanced candidates (e.g., multivalent M protein vaccine, J8-DT) are demonstrating acceptable safety profiles in Phase I/II trials [34,35]. Meanwhile, innovative approaches like conserved virulence factor targeting and live attenuated vectors (e.g., Spy7) may provide broader protection [36,37].

With coordinated efforts through WHO’s GAS Vaccine Roadmap and improved correlates of protection, the field is progressing toward a viable vaccine that meets both immunological and manufacturing requirements for global implementation. Recent breakthroughs in antigen discovery and vaccine delivery systems suggest that licensure of a first-generation GAS vaccine within the current decade is increasingly plausible.

### 3.1. M Protein Vaccines

#### 3.1.1. Multivalent Vaccine

Professor James B. Dale’s team has made seminal contributions to the development of multivalent GAS vaccines. The team first established clinical proof of concept through Phase I trials of recombinant multivalent vaccines, beginning with a 6-valent formulation (2004) that demonstrated safety and robust immunogenicity in healthy adults [38]. This was followed by successful evaluation of a 26-valent vaccine (StreptAvax™) in 2005, further confirming the platform’s clinical feasibility [39]. In the subsequent year, they announced the efficacy of the 26-valent vaccine in a phase II trial [34]. The 30-valent M protein-based vaccine (StreptAnova™) completed phase I trials in 2020, showing excellent tolerability and immunogenicity—critical milestones supporting advanced clinical development [28]. Most recently, preclinical studies demonstrated that their novel 30-valent mRNA vaccine expressing conserved M peptides elicited comparable antibody responses to the protein-based version. This mRNA candidate is currently undergoing phase I clinical evaluation, representing a transformative approach to multivalent Strep A vaccine development [33].

#### 3.1.2. J8-DT Vaccine

In 1997, Professor Michael F. Good’s team first reported the identification of the conserved J8 peptide sequence from the C-repeat region of GAS M protein [40]. Building upon this discovery, researchers subsequently developed the J8-DT (diphtheria toxoid) conjugate vaccine [35]. In 2003, the animal study results demonstrated that this vaccine could effectively induce protective antibody responses in immunized hosts [35]. Subsequently, animal studies demonstrated that the newly designed J8 mucosal vaccine could also elicit protective antibodies [41]. Another independent animal study further confirmed that the J8 epitope was capable of inducing long-term protective antibody responses [42]. In 2018, the phase I clinical trial demonstrated that the J8-DT vaccine exhibited favorable immunogenicity and safety profiles [43].

#### 3.1.3. Others

Currently, although no clinical trial data has been publicly released, several studies have reported that M protein antigens such as J14 [44,45,46], P*17 [47,48], and p145 [49,50,51,52,53] demonstrate potential immunogenicity and safety. And P*17 has entered phase 1 clinical trials [47,48]. We eagerly await the publication of clinical trial data for these candidate antigens.

### 3.2. Non-M Protein Vaccines

So far, no publicly available clinical trial reports exist for vaccines containing only non-M protein antigens without M protein antigens, but numerous animal studies and foundational research have identified some promising candidate antigens. GAS vaccine candidates can be classified into two major categories based on non-M protein antigen localization: surface-exposed proteins and secreted virulence factors. The surface-exposed proteins include adhesion factors (fibronectin-binding protein, streptococcal pili/T-antigen, and lipoteichoic acid), cell wall components (GAS carbohydrate lacking GlcNAc side chain and trirhamnosyl-lipopeptide), as well as enzymes and anchoring proteins (sortase A and C5a peptidase). The secreted virulence factors comprise toxins (streptolysin O and SpeAB fusion protein), metabolic enzymes (arginine deiminase and SpyCEP), and immune modulators (trigger factor-TLR2 complex) (Table 2).

## 4. Health Economics Evaluation and Vaccination Strategy Optimization for GAS Vaccines

Research on the economic evaluation and implementation strategies of GAS vaccines has made significant progress. From a health economics perspective, the development and implementation of GAS vaccines demonstrate substantial socioeconomic value. Scientific cost-effectiveness analysis coupled with tailored immunization strategies can maximize the public health benefits of vaccination.

### 4.1. Health Economics Evaluation

Investigators from the United States, Australia, New Zealand, and other countries have conducted comprehensive health economic assessments of GAS vaccines using different methodologies [11,80,81,82]. Despite variations in approaches, data sources, and publication timelines, their conclusions consistently highlight the substantial health economic benefits of GAS vaccines.

As previously mentioned, the economic and health burdens of GAS diseases are substantial worldwide. In 2018, Jeffrey W. Cannon et al. conducted an economic evaluation of a potential GAS vaccine in Australia. Their analysis revealed that GAS diseases incurred substantial annual burdens: 23,528 disability-adjusted life years (DALYs) and AUD 185.1 million in healthcare costs [82]. In 2021, Jeffrey W. Cannon et al. estimated the economic and health burdens of GAS diseases in New Zealand (NZ). GAS affected 1.5% of the population each year, resulting in an economic burden of NZD 29.2 million and inflicting a health burden of 2373 DALYs [80]. In a 2022 U.S. health economic study of GAS vaccines, Kristin Andrejko et al. estimated the total annual economic burden of invasive and noninvasive GAS diseases at USD 6.08 (95% CI: 5.33–6.86) billion [11]. In 2023, Jung-Seok Lee et al. estimated the global economic burden of GAS-related diseases, revealing substantial variations across income groups [81]. These findings demonstrate the significant economic impact of GAS infections and underscore the critical need for effective preventive measures, particularly vaccine development, to reduce this global health burden.

Under such severe circumstances, the role of vaccines in health economics becomes particularly prominent. One study demonstrated that vaccine implementation could provide significant value across different age groups, with varying effectiveness against specific infections [82]: For infant vaccination (cost-effective at AUD 260 per course), prevention of throat infections (30%), skin infections (33%), and cellulitis (28%) contributed most to the vaccine’s value. For child vaccination (AUD 289 per course), these proportions were 47%, 26%, and 22%, respectively. For adult vaccination (AUD 489 per course), the value distribution shifted markedly to 2%, 15%, and 74%. Another modeling study [11] evaluated vaccines meeting WHO Preferred Product Characteristics (providing 6-year protection). The analysis demonstrated that pediatric vaccination (administered at 12 + 18 months) could avert USD 609 million (95% CI: 558–663) annually; adding a booster dose at 5 years would increase the annual savings to USD 869 million (95% CI: 798–945); elderly vaccination (at age 65+) could prevent USD 326 million (95% CI: 271–387) yearly in costs associated with invasive GAS disease. Preventing GAS diseases would have substantial economic and health benefits globally.

### 4.2. Vaccination Strategy Optimization

The WHO GAS Vaccine Development Roadmap establishes key implementation parameters, prioritizing equitable allocation [83]. The primary vaccination series targets infants/young children (≤3 doses), though optimal timing requires further epidemiological validation—particularly whether to administer in early infancy or through staged early childhood dosing [83]. Critical evidence gaps remain regarding (1) primary series dosing schedules and (2) booster requirements at key life stages (e.g., school entry, adolescent transition, pregnancy, or elderly). These strategic considerations align with disease burden peaks, where multiple boosters may prove both necessary and operationally feasible. Moreover, we could prioritize allocating limited resources to develop a relatively effective and safe GAS vaccine first, followed by continuous optimization and refinement. The near-term objective focuses on demonstrating candidate vaccine safety and efficacy against pediatric GAS pharyngitis and skin infections [83]. Building upon this foundation, the long-term vision aims to develop a globally accessible vaccine that prevents the full spectrum of GAS manifestations—from acute infections (including pharyngitis, skin infections, cellulitis, and invasive disease) to secondary immune-mediated sequelae (particularly kidney disease, rheumatic fever, and rheumatic heart disease)—while simultaneously reducing antibiotic use and associated mortality.

## 5. Accelerating the Development and Approval of GAS Vaccines

Accelerating the development and approval of GAS vaccine requires a multifaceted approach involving scientific, regulatory, and logistical strategies. Below are key measures that could expedite the process.

### 5.1. Enhanced Funding and Global Collaboration

**Figure 2:**
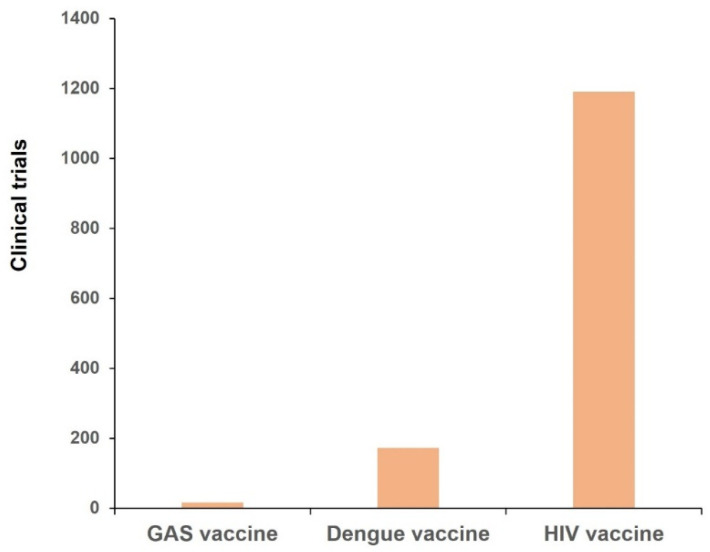
Comparative number of registered vaccine clinical trials: GAS vs. dengue virus vs. HIV.

Increased financial support and international collaboration are very important for the GAS vaccines. Governments, philanthropic organizations, and global health institutions should provide sustained funding to support research, clinical trials, and manufacturing scale-up. As of 27 April 2025, statistics from the ClinicalTrials.gov official website show that the number of registered vaccine-related clinical trials for GAS is significantly lower than those for dengue virus and HIV [84,85,86] (Figure 2). This indirectly indicates that vaccine development efforts for GAS need to be substantially increased. Investment in a Strep A vaccine could create enormous benefits for comparatively little cost. It represents one of the highest return uses of public spending. Policy can promote Strep A vaccine development through direct funding of projects and by promoting financial mechanisms that allow the private sector to diversify its research and development investment [87]. Additionally, global partnerships among academia, industry, and public health organizations should be established to share resources, data, and expertise. Initiatives like the *Streptococcus pyogenes* Vaccine Global Consortium (SAVAC) can play a pivotal role [88].

### 5.2. Leverage Advanced Vaccine Technologies

With the advancement of novel vaccine technologies—such as mRNA vaccines and next-generation adjuvants—the development of GAS vaccines has gained some momentum. However, judging by the current number of clinical trials and their phases, this acceleration still needs to be improved. New breakthroughs in vaccine design and faster progress in clinical trials are essential to bring a viable GAS vaccine to market as soon as possible [87]. Additionally, we can still draw lessons from the development path of pneumococcal vaccines, such as key antigen discovery breakthroughs, advances in polysaccharide-protein conjugate technology and serotype coverage strategy [89,90]. In summary, accelerating technological breakthroughs is another critical factor in achieving the early commercialization of a GAS vaccine.

### 5.3. Streamline Clinical Trials

Adaptive trial designs that allow for simultaneous evaluation of multiple vaccine candidates should be implemented, reducing the time needed to identify promising candidates [91,92], and regulatory alignment across regions to streamline trial approvals and vaccine licensure processes should be fostered. Expedited review pathways, such as the FDA’s Fast Track designation [93,94], could be leveraged.

### 5.4. Increase Advocacy and Public Awareness

To address the substantial global burden of GAS-related morbidity and mortality, accelerating vaccine development as a public health priority must go hand-in-hand with proactive community engagement. It boosts clinical trial participation to accelerate development timelines, counters harmful misinformation that could disrupt research, increases political will for funding through greater public awareness, and provides valuable feedback to help scientists prioritize research directions that align with community needs [95,96,97,98,99]. The HPV vaccine saw higher uptake after awareness campaigns corrected misconceptions [100,101]. COVID-19 vaccines benefited from global efforts like the WHO’s “Vaccine Equity” campaign [102,103,104]. By increasing advocacy and awareness, we can accelerate vaccine development, ensure widespread adoption, and save lives through science and solidarity.

By implementing these strategies, the development and approval timeline for GAS vaccines could be significantly shortened, bringing life-saving interventions to those in need more quickly.

## 6. New Avenues for Future Research

### 6.1. Novel Insights into the Infection Spectrum, Disease Spectrum, and Pathogen Profile

The infection spectrum of GAS is undergoing dynamic changes, as evidenced by recent developments such as the resurgence of scarlet fever and the impact of the COVID-19 pandemic on GAS epidemiology. These factors have inevitably reshaped the GAS disease landscape, and their implications for vaccine efficacy and accessibility require further investigation. Additionally, it is currently widely believed that GAS is primarily transmitted through respiratory droplets and contact. However, in specific settings such as schools, which mode of transmission is more likely to occur? Furthermore, how do mucosal vaccines and injectable vaccines differ in their effects on the pathogenicity and transmissibility of GAS? These questions have significant implications for the future development and approval of vaccines.

Recent research has identified several novel complications associated with GAS infections, including neuropsychiatric manifestations like pediatric autoimmune neuropsychiatric disorders (PANDAS/PANS) [105,106,107,108] and cardiovascular sequelae such as post-infectious myocardial fibrosis [109]. Meanwhile, evidence suggests an association between GAS infection and psoriasis [110]. Critical knowledge gaps remain regarding vaccine efficacy against atypical complications.

In recent years, emerging trends in GAS pathogens have been observed, including the spread of the M1UK strain [111,112,113,114] and reports of penicillin-resistant virulent variants [115]. Whether these epidemic strains and drug-resistant variants exhibit enhanced immune evasion has critical implications for the future efficacy of vaccines.

To advance GAS vaccine development, critical research priorities include (1) refining global disease burden estimates through enhanced epidemiological surveillance; (2) characterizing the full spectrum of GAS infection dynamics and natural disease progression; (3) elucidating mechanisms of secondary immune-mediated sequelae (particularly rheumatic heart disease pathogenesis); and (4) quantifying the antibiotic stewardship potential of vaccination by assessing its impact on antimicrobial usage patterns and resistance-related outcomes. These interconnected investigations will establish the foundational evidence required for vaccine implementation strategies.

### 6.2. Exploration of Novel Antigens, Innovative Technologies, and Next-Generation Vaccine Platforms

Although we have identified a series of GAS candidate antigens, we have not yet achieved the goal of developing a viable GAS vaccine. We must not halt the discovery of new potential antigens—on the contrary, we must intensify efforts to discover and design safer, more effective GAS antigens by integrating next-generation technologies including AI-enhanced genome mining for conserved antigen discovery [116,117,118], Cryo-EM guided epitope focusing [119,120], computational antigen engineering, and multivalent chimeric antigen design [121,122,123].

We have been continuously exploring whether the technologies supporting vaccine development and production can be further innovated—such as respiratory mucosal vaccine technology (nanoparticle-encapsulated intranasal formulations [124,125], M-cell targeting bioconjugates [126]), non-invasive vaccine delivery systems (dissolvable microneedle arrays [127,128], electroporation-assisted DNA vaccination [129,130]), and novel adjuvant platforms. These advancements would significantly enhance the accessibility and efficacy of GAS vaccines (Table 3).

Finally, we must proactively apply cutting-edge vaccine technologies to GAS vaccine development, particularly nucleic acid-based platforms (including mRNA-LNP constructs encoding multiple virulence factors [131,132] and self-amplifying RNA systems [133,134]) and live vector delivery systems (such as attenuated Salmonella typhi vectors [135,136] and rhabdovirus-based delivery platforms [137,138]) (Table 3).

## 7. Conclusions

GAS infection is a major global public health issue, with a high disease burden that particularly endangers children and adolescents. Since 2011, there has been a resurgence of cases, including STSS outbreaks in Japan, reminding us that we must urgently put an end to the devastation caused by GAS. Currently, aside from diagnosis and treatment, there are no effective preventive measures against GAS—meaning no approved vaccines yet. Existing vaccine development efforts primarily focus on M protein-based vaccines (multivalent, J8-DT, J14, P*17, and p145), along with the discovery of some non-M protein antigens as potential candidates. However, so far, progress has not advanced beyond phase 2 clinical trials, leaving a long way to go before large-scale deployment. We can draw valuable lessons from the development of COVID-19 and pneumococcal vaccines. To accelerate the availability of GAS vaccines, we must increase investment in research and development, foster broader collaborations, leverage advanced vaccine technologies, streamline clinical trials, and enhance advocacy and public awareness—we can no longer afford to wait. The world must unite to make GAS vaccines a reality and end the suffering of children and adolescents at the hands of this devastating pathogen.

## Figures and Tables

**Table 1:** GAS M protein-based candidate vaccines in clinical trials.

Type	Vaccine Target Region of M Protein	Clinical Trial Phase	Developers
6-valent vaccine	the N-terminal	Phase I	James B Dale’s team
26-valent vaccine	the N-terminal	Phase II	James B Dale’s team
30-valent vaccine	the N-terminal	Phase I	James B Dale’s team
Novel 30-valent mRNA vaccine	the N-terminal	Phase I	James B Dale’s team
J8-DT Vaccine	the C-repeat	Phase I	Michael F. Good’s team
P*17 vaccine	the C-repeat	Phase I	Vanessa Meier-Stephenson’s team

**Table 2:** GAS non-M protein vaccine candidate antigens.

Cellular Localization	Biological Function	Antigens	Protective Mechanism/Evidence
Surface-Exposed Proteins	Adhesion Factors	Fibronectin-binding protein (Sfb1)	Protection against lethal GAS challenge, bacterial attachment prevention, and colonization inhibition [54,55,56,57]
		Streptococcal pili/T-antigen	Anti-adhesion neutralizing activity [58,59,60]
		Lipoteichoic acid (LTA)	Anti-adherence activity to pharyngeal epithelium by GAS [61]
	Cell Wall Components	GAS carbohydrate/lacking GlcNAc side chain	Experimentally induced passive protection in mice [62,63]
		Trirhamnosyl-lipopeptide	75–97% opsonic activity against GAS clinical isolates comparable to J8-lipopeptide subunit vaccine [64]
	Enzymes and Anchors	Sortase A	Nasal-associated lymphoid tissue colonization suppression [65]
		C5a peptidase (ScpA)	Immune evasion protease neutralization [66,67,68,69,70]
Secreted Virulence Factors	Toxins	Streptolysin O (SLO)	Neutralization of SLO-mediated hemolysis [71,72]
		SpeAB fusion protein	Superantigen-neutralizing antibody response induction [73,74,75]
	Metabolic Enzymes	Arginine deiminase (ADI)	Multi-serotype GAS immunity induction [76,77]
		SpyCEP (IL-8 protease)	Multi-serotype GAS protection in animal models [68,78]
	Immune Modulators	Trigger factor (TF)-TLR2	Protective immunity elicitation [77,79]

**Table 3:** Advantages and limitations of key technologies enabling GAS vaccine development.

Technology	Advantages	limitations
Respiratory Mucosal Vaccines	Induces mucosal immunity	Potential immune tolerance issues
	Needle-free administration improves compliance	Precise delivery requirements
	Rapid pathogen blockade at entry site	Stability challenges
Novel Adjuvant Platforms	Enhances immunogenicity	Risk of excessive immune activation
	Enables antigen dose sparing	May cause respiratory irritation
mRNA-LNP	Rapid development	Low delivery efficiency in certain tissues
	Non-integrating and safe	Requires booster doses
	Encodes multiple antigens	Cold chain dependency
Self-Amplifying RNA	Ultra-low dose efficacy	Large molecular size requires optimized delivery
	Prolonged antigen expression	Potential innate immune suppression of translation
	Built-in adjuvant effect via dsRNA intermediates	Viral-derived components may raise safety concerns
Live Vector Systems	Mimics natural infection	Replication-competent vectors risk virulence reversion
	Multivalent antigen capacity	Complex manufacturing increases costs
